# Suspension sutures facilitate single-incision laparoscopic-assisted rectal pull-through for Hirschsprung disease

**DOI:** 10.1186/s12893-021-01260-w

**Published:** 2021-05-31

**Authors:** Liem Thanh Nguyen, Anh Tho Nguyen, Quang Thanh Nguyen, Quynh Anh Tran, Hau Duc Bui, Hien Duy Pham

**Affiliations:** 1 Vinmec International Hospital, 458 Minh Khai, Vinh Tuy, Hai Ba Trung, Hanoi, Vietnam; 2 Vietnam National Children’s Hospital, Hanoi, Vietnam

**Keywords:** Hirschsprung disease, Laparoscopic, Single incision, Suspension sutures, Rectal pull-through

## Abstract

**Background:**

To present a surgical technique of single-incision laparoscopic-assisted endorectal pull-through (SILEP) with suspension sutures using conventional instruments for Hirschsprung disease (HD) and its long-term follow-up outcomes.

**Methods:**

The procedure began with a 1 cm transumbilical skin incision. Three separate punctures were made in the fascia with a 5 mm scope in the middle and 5 mm and 3 mm ports for working instruments on the left and right, respectively. The first suspension suture was placed to secure the sigmoid colon to the abdominal wall. A window was created through the rectal mesentery, and dissection around the rectum was carried out. The second suspension suture was performed to suspend the rectovesical peritoneal fold or the rectovaginal peritoneal fold to the abdominal wall. Dissection around the rectum was continued downward to approximately 1 cm below the peritoneal fold. Then, the operation was completed by a transanal approach.

**Results:**

Forty patients underwent SILEP from March 2013 to April 2015. The median age was 2.7 months (ranging from 1 to 17 months). The mean operative time was 96 ± 23 min. No conversion to an open operation was required. The average hospitalization time was 4.5 ± 2 days. There were no intraoperative or perioperative complications. Long-term follow-up results were obtained from 38 patients. A frequency of defecation from every other day to twice a day was noted for 33 patients (86.8%) and more often for 5 patients (13.2%). Two patients had enterocolitis (5.2%).

**Conclusion:**

Single-incision laparoscopic rectal pull-through with suspension sutures using conventional instruments is feasible and safe for HD with good long-term outcomes.

## Background

HD is conventionally managed by an open, staged approach, which results in multiple unpleasant scars. In 1995, Georgeson first introduced a laparoscopic rectal pull-through for HD [1]. Since then, this approach has become a routine procedure in many centers and has provided good outcomes. The conventional laparoscopic operation is usually performed using four incisions in the abdominal wall [2–7]. To achieve a better cosmetic result, in 2010, Muenstere et al*.* described the single-incision laparoscopic endorectal pull-through (SILEP) for Hirschsprung disease [8]. This approach has been applied in some centers with moderate success [9–12]. Randomized studies demonstrated that the short-term postoperative results of SILEP did not differ from those of conventional laparoscopic surgery whereas the SILEP group had better cosmetic results [10, 11]. However, technical difficulties in performing the operation impedes its wider popularity, with only a small number of publications. We hypothesize that those technical difficulties may be minimized by introducing suspension sutures. Before 2013, we performed laparoscopic operations for HD using three or four incisions [2]. To improve the feasibility of the operative maneuvers, we have performed a modified SILEP for HD at the National Children’s Hospital of Vietnam since March 2013.

This report aims to describe our modified procedure and its long-term outcomes for HD.

## Patients and methods

### Patients

A series of consecutive patients with HD who underwent SILEP with two suspension sutures during the study period was selected. The diagnosis was initially based on the clinical manifestations and colonography (typical image included a distal narrowed zone, a transition zone and a dilated colonic segment) and later confirmed with an intraoperative frozen section biopsy.

*Inclusion criteria* The patient’s age was from 1 to 24 months. The aganglionic segment was located in the rectum and sigmoid colon.

*Exclusion criteria* Patients with conditions such as previous colostomy, previous peritonitis, and previous abdominal operations were excluded.

### Methods

A prospective study was performed from March 2013 to April 2015, and postoperative follow-up was continued until February 2020.

The rate of conversion to an open operation, operative time, and perioperative and late complications were recorded. The Rintala scoring system was used to evaluate intermediate and long-term outcomes of fecal continence. This system includes seven factors: ability to hold back defecation, feels/reports the urge to defecate, frequency of defecation, soiling, accidents, constipation, and social problems. Each factor is scored from 0 to 3, except for the frequency of defecation, which is scored from 1 to 2. The outcome was classified as excellent when patients had a score of 18 to 20 points. The patients who scored 9–16 points were considered to have good results with occasional staining and infrequent accidents. Patients who had a score of 7–11 points were classified as having fair results with intermittent daily soiling or staining. Patients who had a score of 6–9 points were considered poor results. They had to use daily enemas due to severe constipation or had constant soiling [13].

The Manchester scoring system consisting of five factors was used to evaluate the cosmetic appearance of the scars. Each factor was scored from one to four points. The total score ranges from 5 to 28 points, and lower scores indicate a better cosmetic appearance [14].

### Operative technique

**Fig 1:**
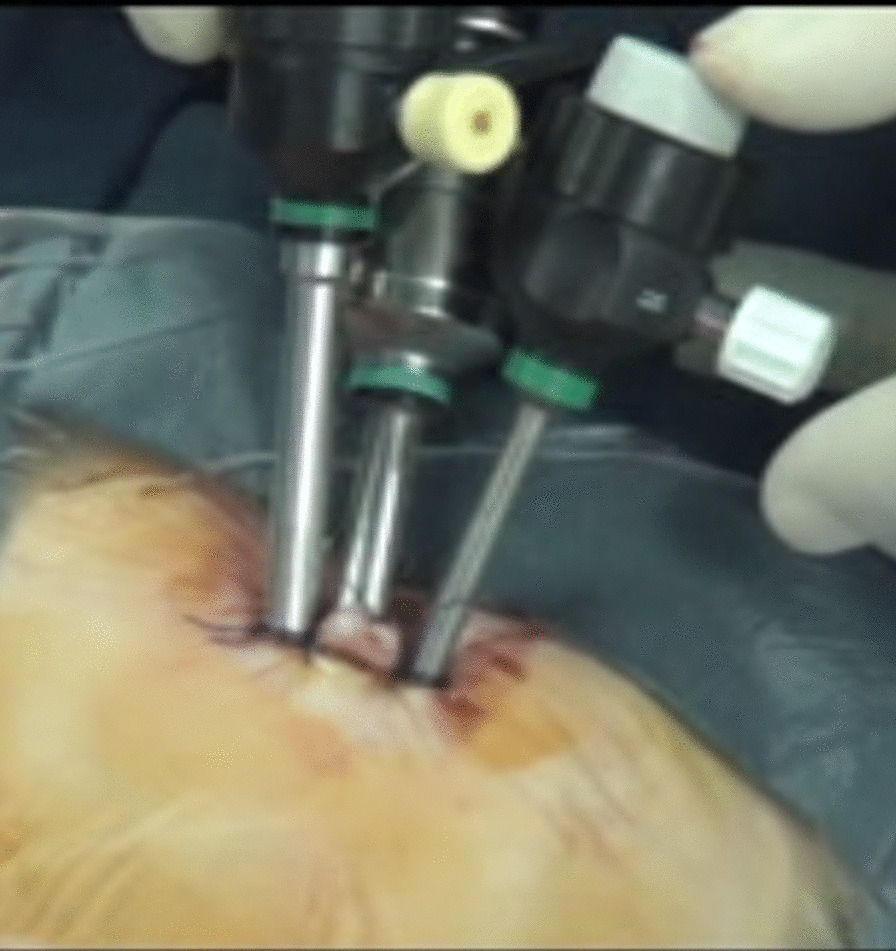
Trocar’s placement

The procedure began with a 1 cm transumbilical skin incision. Three separate punctures were made into the fascia for a 5 mm conventional scope in the middle and 5 mm and 3 mm ports for working instruments on the left and right sides, respectively. Conventional laparoscopic instruments were used to perform the operation (Fig. 1).

Extramucosal biopsies were performed on the narrow segment (suspected aganglionic segment) and the dilated segment (presumed healthy colon).

**Fig 2:**
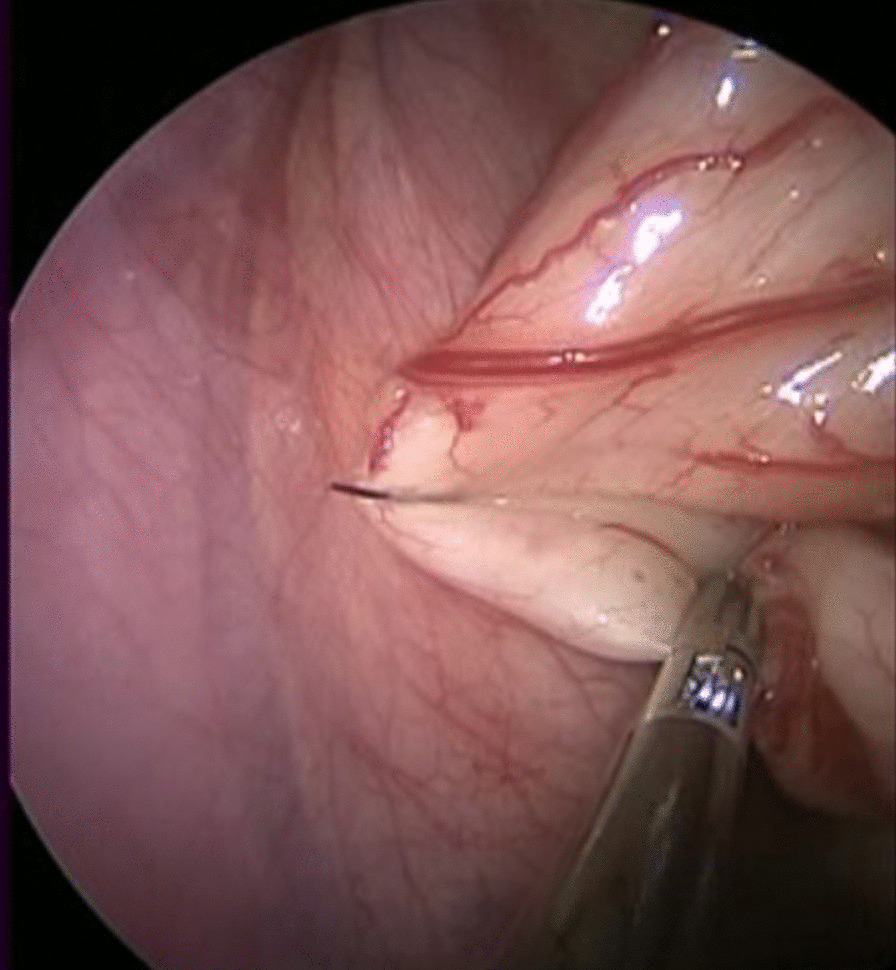
Secure the sigmoid to the abdominal wall

**Fig 3:**
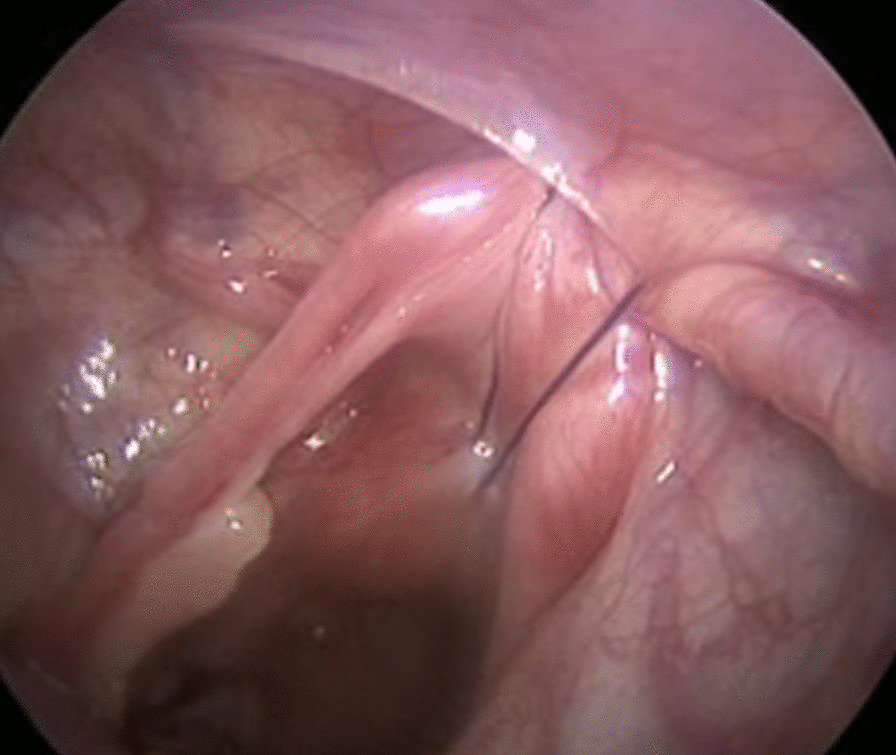
Suspend the peritoneal fold to the abdominal wall

The first suspension suture was performed to secure the sigmoid colon to the abdominal wall (Fig. 2). A window was created through the rectal mesentery, and dissection around the rectum was carried out. The second suspension suture was performed to suspend the rectovesical peritoneal fold or the rectovaginal peritoneal fold to the abdominal wall (Fig. 3). Dissection around the rectum was continued downward to approximately 1 cm below the peritoneal fold.

Dissection of the colonic mesentery was continued upward to provide more length to facilitate the tension-free mobilization of the colon through the anus. Laparoscopy was then stopped, and the remaining operative intervention was performed trans-anally as described in the previous publication [15].

Fifteen days after the surgery, anal dilation was started once a day for one month and then twice per week for the next two months. Dilatator’s size varied from number 12th to number 15th based on patient’s age [16].

## Results

Our study involved a total of 40 patients, including 38 boys and 2 girls. The median age was 2.7 months (ranging from 1 to 17 months). Twenty-one patients (55.5%) were ≤ 3 months old, and 19 patients (45.5%) were over 3 months old. Combined malformations included Down syndrome in 2 patients, atrial septal defect in 1 patient, ventricular septal defect in 1 patient, and ureteral duplication in 1 patient.

Location of the aganglionic segments: rectum in 29 patients (72.5%) and sigmoid colon in 11 patients (27.5%). The total operative time varied from 50 to 150 min (mean: 96 ± 23 min). The length of the resected colon ranged from 15 to 40 cm (mean: 19.6 ± 6.2 cm). Insignificant blood loss was recorded. No conversion to open surgery was required. There was no perioperative complication. The average postoperative hospitalization time was 4.5 ± 2 days.

### Intermediate follow-up results

Intermediate follow-up with a mean duration of 23.2 ± 9.6 months (ranging from 15 to 42 months) was obtained for all 40 patients whose age varied from 17 to 55 months (mean: 27.7 ± 10.8 months). Temporary perianal dermatitis occurred in 6 children (15%) in the early period after discharge. Thirty-nine children obtained spontaneous defecation, whereas constipation persisted in one patient who required a second operation to remove a residual aganglionic segment. The outcomes of fecal continence according to the Rintala scoring system are presented in Table 1.

**Table 1:** Evaluation of fecal continence according to Rintala scoring system

Factor	Intermediate	Long-term
	N (%)	N (%)
*Ability to hold back defecation*	40 patients	38 patients
Always	32 (80%)	35 (92.1%)
Problems less than 1/week	4 (10%)	3 (7.9%)
Weekly problem	4 (10%)	0
No voluntary control	0	0
*Feel/reports the urge to defecate*	40 patients	38 patients
Always	35 (87.5%)	35 (92.1%)
Most of time	5 (12.5%)	3 (7.9%)
Uncertain	0	0
Absent	0	0
*Frequency of defecation*	40 patients	38 patients
Every other day to twice a day	28 (70%)	33 (86.8%)
More often	11 (27.5%)	5 (13.2%)
Less often	1 (2.5%)	0
*Soiling*	40 patients	38 patients
Never	32 (80%)	38 (100%)
Staining less than 1/week, no change of underwear required	8 (20%)	0
Frequent staining, change of underwear often required	0	0
Daily soiling, requires protective aids	0	0
*Accidents*	40 patients	38 patients
Never	36 (90%)	37 (97.4%)
Fewer than 1/week	4 (10%)	1 (2.6%)
Weekly accident; often requires protective aids	0	0
Daily, required protective aids during day and night	0	0
*Constipation*	40 patients	38 patients
No constipation	31 (77.5%)	38 (100%)
Manageable with diet	8 (20%)	0
Manageable with laxatives	0	0
Manageable with enemas	1 (2.5%)	0
*Social problems*	40 patients	38 patients
No social problems	36 (90%)	38 (100%)
Sometimes (Foul odors)	4 (10%)	0
Problem causing restriction in social life	0	0
Severe social and/or psychological problems	0	0

Enterocolitis (the presence of abdominal distension, explosive diarrhea with foul-smelling or bloody stools that required antibiotics in the last six months) was noted for 5 patients (12.5%), and soiling occurred in 8 patients (20%). Anastomotic stenosis or fistula was not observed in any patient.

### Long term follow-up results

The mean follow-up was 65 ± 9.9 months (ranging 56 to 84 months), which was achieved in 38 patients (95%). The patient’s age at the evaluation varied from 58 to 96 months (mean: 69.1 ± 10.9).

Enterocolitis occurred in 2 patients (5.3%). Soiling or constipation was not observed in any patient. Urinary continence was noted in 38 children. Among them, male children had a normal penile erection according to their parents’ observations. All patients were classified as having excellent results according to the Rintala scoring system for fecal continence (Table 1).

### Clinical scar assessment

All patients achieved 5 points on Manchester scoring system for clinical scar assessment. Satisfaction with surgical scar was also confirmed in all parents.

## Discussion

In HD with a rectosigmoid form, the sigmoid colon is often dilated, which can reduce the operative field and lead to technical difficulties when the operation is performed using a single incision. Our suspension sutures can clearly expose the surgical site and greatly enhance the feasibility of SILEP. The sigmoid and rectal mesentery can be easily visualized by making a suspension suture that attaches the dilated sigmoid colon to the abdominal wall. Dissection of the mesenteries can, therefore, be achieved with ease. Another technical challenge usually encountered is the separation of the rectum from the bladder or the vagina. This can also be facilitated by making a second suspension suture from the peritoneal reflection onto the abdominal wall. Thus, a wider space between the bladder or the vagina and the anterior wall of the rectum is made available for precise dissection.

Our results have demonstrated that SILEP with our modifications is feasible and safe for HD. The operation was successfully performed in all patients without conversion to open surgery. Blood loss was nonsignificant. No perioperative complications were observed.

To the best of our knowledge, our mean operating time of 95 min is the shortest among those reported in the literature (Table 2) [8, 10–12]. We believe the suspension sutures have facilitated dissection and, in turn, reduced the operating time.

**Table 2:** Operative time according to different publications

Publications	Mean operative time (min)
Muenster OJ et al*.* [8]	145 ± 44
Tang ST et al*.* [10]	122 ± 18
Xia X et al*.* [11]	226 ± 69.4
Meng X et al*.* [12]	211.91 ± 53.62
This study	96 ± 23

In long-term follow-up, the incidence of anastomotic stenosis, enterocolitis, soiling, fecal incontinence, and constipation in our series was much lower than that in other studies using laparoscopic transanal pull-through (Table 3).

**Table 3:** Long-term outcomes between different reports

Authors	Anastomotic stricture	Enterocolitis (%)	Soiling (%)	Fecal incontinence (%)	Constipation (%)
Tang ST (2012) [3]	2.2	7.7	3.6	*	1.6
Tomuschat C (2016) [17]	*	9.14	**	11.14	11.14
Zheng Z (2018) [18]	9	17.9	15.6	0	6.4
Meng X (2020) [12]	*	18.34	13.76	3.67	17.43
Liem NT	0	5.2	0	0	0

^*^No information

^**^Number of soiling and fecal incontinence not separated

Our results demonstrated that the longer the follow-up, the better the outcomes. Enterocolitis was reduced from 12.5% in the intermediate follow-up to 5.2% in the long-term follow-up. The frequency of defecation from every other day to twice a day increased from 70% in intermediate follow-up to 87% in long-term follow-up. The incidence of staining/soiling from 20% in the intermediate follow-up was reduced to 0% in the long-term follow-up.

Regarding the manifestations of surgical scars, all patients in our study achieved the best scores according to the Manchester scoring system. This result proves that SILEP provides a high aesthetic value.

## Conclusion

SILEP with two suspension sutures is feasible, safe, and provides excellent cosmetic results and satisfactory long-term outcomes of fecal continence for HD.

## Data Availability

The data are available from the corresponding author on reasonable request.

## Abbreviations

SILEP: Single incision laparoscopic-assisted endorectal pull-through
HD: Hirschsprung disease
